# Identifying a novel PHOX2B gene variant in a neuroblastoma family: A case report

**DOI:** 10.1016/j.heliyon.2024.e26581

**Published:** 2024-02-19

**Authors:** Xiongwei Wu, Wenli Xiu, Na Zhou, Jingli Zhang, Xiwei Hao, Qian Dong

**Affiliations:** Department of Pediatric Surgery, Affiliated Hospital of Qingdao University, Qingdao, China

**Keywords:** Neuroblastoma, Mutation, Exon, Sequencing, Case report

## Abstract

Neuroblastoma is a childhood cancer characterized by the formation of tumors derived from neuroblasts. Identifying the genetic mutations underlying neuroblastoma for genetic counseling and early diagnosis is essential. Thus, this study aimed to screen for pathogenic gene variants within a neuroblastoma family, aiming to contribute to genetic counseling practices. Clinical data was collected from a family affected by neuroblastoma, and peripheral blood DNA samples were obtained from all family members. A combination of whole-exome sequencing and Sanger sequencing was utilized to detect potential gene mutations. Proband 1 and her sister (Proband 2) were diagnosed with neuroblastoma, while their parents and siblings were unaffected. The analysis revealed a novel missense mutation, c.422G > A (p.Arg141Gln), in the PHOX2B gene, which was inherited from the mother. Notably, this mutation represents a previously unreported variant within the PHOX2B gene. Detecting the missense mutation c.422G > A (p.Arg141Gln) in the PHOX2B gene implies its potential pathogenic role within this neuroblastoma family. This finding widens the range of mutations observed in the PHOX2B gene and has important implications for early neuroblastoma diagnosis within this family.

## 1 Introduction

Neuroblastoma (NB) is childhood's most common extracranial solid tumor originating from the adrenal medulla or paravertebral sympathetic ganglia. It accounts for 15% of pediatric cancer-related deaths [1]. NB typically presents with symptoms such as abdominal pain or a palpable mass, diagnosed through imaging studies and biopsy. Understanding the genetic basis of NB is crucial for accurate diagnosis and treatment. While most neuroblastomas are sporadic, occurring randomly, approximately 1–2% of cases exhibit autosomal dominant inheritance within families [2]. Familial neuroblastoma often presents earlier and has a higher risk of bilateral tumors than sporadic cases. Understanding the genetic factors underlying familial NB can provide valuable insights into disease mechanisms.

Significant progress has been made in elucidating the genetic basis of neuroblastoma susceptibility. Studies have established anaplastic lymphoma kinase (ALK) [3] and PHOX2B [4] as significant susceptibility genes in familial neuroblastoma cases. ALK gene mutations drive the abnormal growth of neural crest cells, while mutations in the PHOX2B gene disrupt normal sympathetic nervous system development. Furthermore, candidate genes such as NBAT-1 [5], BARD1 [6], and TP53 [7] have been identified as independent driving factors for sporadic neuroblastoma susceptibility, playing roles in both tumor development and the maintenance of the oncogenic phenotype. These genes involve cellular processes, including cell growth regulation and DNA repair.

The main objective of this study is to investigate neuroblastoma families and identify potential pathogenic gene variants, aiming to provide more accurate genetic counseling and early diagnosis for affected patients.

## 2 Case presentation

**Fig. 1:**
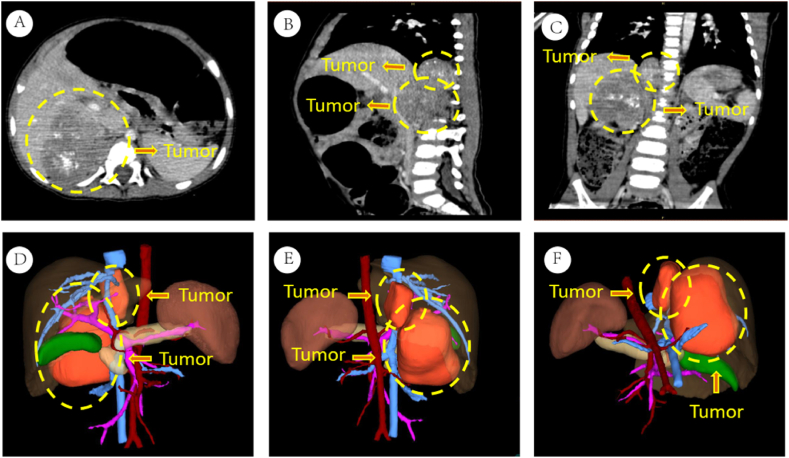
Presents the imaging data of proband 1. Figures A–C display abdominal CT images. Figures D–F show three-dimensional imaging pictures.

**Fig. 2:**
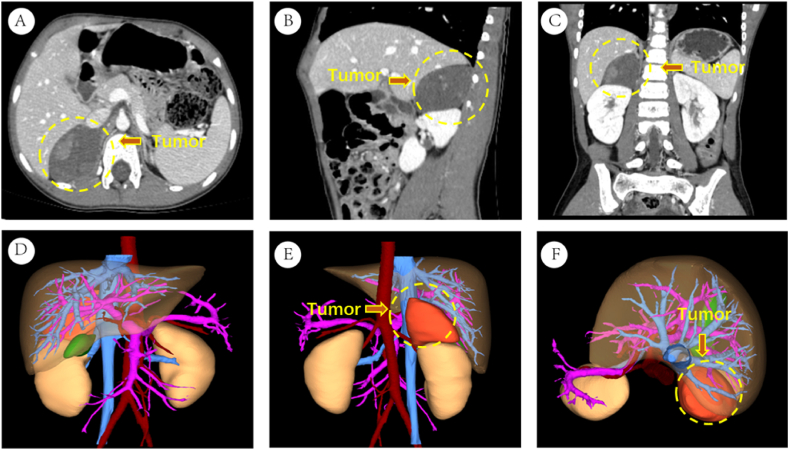
Presents the imaging data of proband 2. Figures A–C display abdominal CT images. Figures D–F show three-dimensional imaging pictures.

The family members in this pedigree consist of the parents (father and mother) and their three children (proband 1, proband 2, and their older sister). Proband 1, a 1-year-old male, presented himself at the hospital with complaints of abdominal distension that had lasted for one week. Abdominal CT imaging revealed a mass measuring 32mm*18mm, with a mixture of high and low density near the right lower spine, as well as another group measuring 42mm*56mm, with a mix of high and low density in the right adrenal gland area (Fig. 1A–F). Blood biochemistry analysis revealed a neuron-specific enolase level of 74.45ng/ml (reference range: 0–17ng/ml). Five days after confirming the diagnosis, he underwent surgery. Pathological examination identified a neuroblastoma in the right adrenal gland area and a ganglioneuroblastoma adjacent to the right lower spine. Proband 2, the older sister of the proband, also had a relevant medical history. At the age of 6, she experienced continuous vomiting for one week. Abdominal CT examination revealed a mass, approximately 46mm*57mm, with a high and low-density mixture in the right adrenal gland area. Blood biochemistry analysis indicated a neuron-specific enolase level of 17.34ng/ml (reference range: 0–17ng/ml). Proband 2 underwent surgical resection, and the pathological examination confirmed the diagnosis of neuroblastoma (Fig. 2A–F). The parents of the probands were not blood relatives, and regular medical check-ups showed no abnormal results for the probands' father, mother, and older sister.

**Fig. 3:**
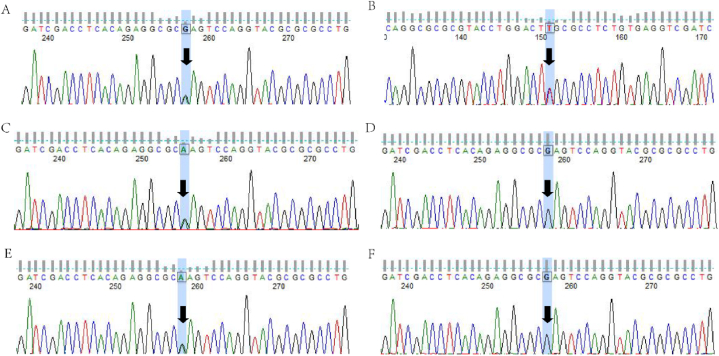
The Sanger sequencing validation results for the proband and their family members. A and B represent the forward and reverse sequencing results of proband 1, respectively. C represents the forward sequencing result of the mother, while D represents the bold sequencing result of the father. E represents the sequencing result of proband 2, and F represents the sequencing result of the older sister.

**Fig. 4:**
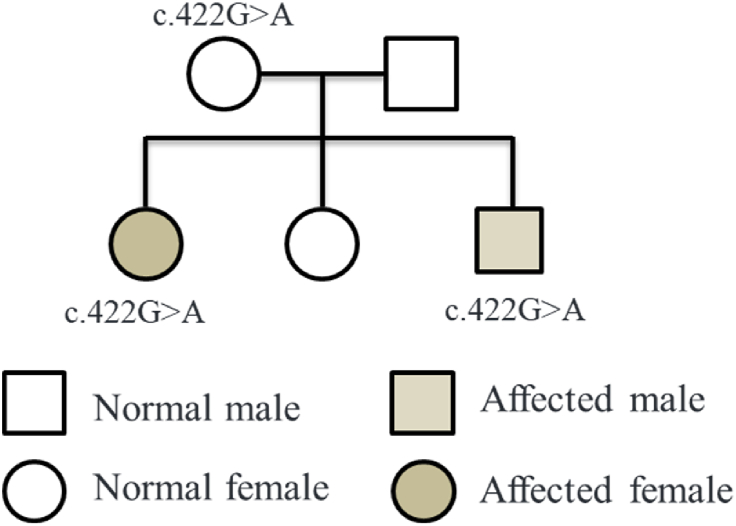
Pedigree chart.

To screen for pathogenic genes in this neuroblastoma pedigree, venous blood samples were collected from all pedigree members for whole-exome analysis. Following comparison and filtering against databases, proband 1 and proband 2 carried a missense mutation c.422G > A (p.Arg141Gln) in the PHOX2B gene. The Sanger sequencing results for proband 1 and proband 2 were consistent with the whole exome sequencing results (Fig. 3A–F). The mother was found to be a carrier of the heterozygous mutation c.422G > A, while the father and their older sister did not carry this mutation. Therefore, the variants in the two cases were inherited from the mother. We constructed a pedigree char (Fig. 4).

**Fig. 5:**
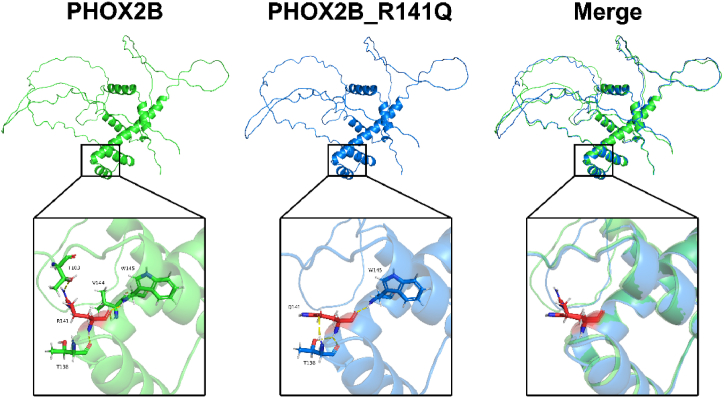
3D modeling of the mutated PHOX2B protein encoded by it using computer simulation.

The bioinformatics analysis revealed that the missense mutation c.422G > A in the PHOX2B gene resulted in a change from arginine (Arg) to glutamine (Gln) at the corresponding amino acid position. We searched for the mutation frequency of this locus in the 1000 Genomes Project and found no information on it, which may indicate that this mutation is rare or absent in the healthy population. Computer simulations were performed to model the mutant PHOX2B protein and compare it with the wild-type protein structure. The analysis showed that the mutation caused structural changes in specific alpha helices within the secondary structure of the PHOX2B protein, leading to a spatial alteration in the peptide chain. This alteration may potentially impact the biological function of the PHOX2B protein (Fig. 5).

## 3 Discussion and conclusion

Neuroblastoma is a highly malignant and deadly tumor originating from the sympathetic nervous system's precursor or immature cells. Approximately 1–2% of neuroblastoma cases are inherited in an autosomal dominant manner, often associated with neurocristopathies such as congenital central hypoventilation syndrome and Hirschsprung disease [8]. The PHOX2B gene encodes a protein with homologous domains and is the first gene associated with genetic susceptibility in neuroblastoma patients [9].

Mutations in the PHOX2B gene, particularly within the alanine repeat sequences, correlate with clinical phenotypes' severity [10]. Furthermore, it plays a crucial role in autonomic ganglia development in the autonomic nervous system [11]. In neuroblastoma, lineage-specific PHOX2B mutations promote malignant transformation during terminal neuroblastic differentiation. Another significant driver gene in familial neuroblastoma is anaplastic lymphoma kinase (ALK), and PHOX2B drives the expression of the ALK gene by directly binding to its promoter [12].

Children with PHOX2B variants show a solid correlation between genotype and phenotype [13], with missense, frameshift, or truncating mutations more predisposed to neuroblastoma susceptibility [14,15]. In the presented family, both proband 1 and proband 2 are affected by neuroblastoma, while their parents and another sibling are healthy individuals. The genetic analysis determined they carry a missense mutation c.422G > A in the PHOX2B gene. Structural changes in the three-dimensional conformation of the mutant PHOX2B protein were observed through 3D modeling, and Research has shown that this variant significantly impairs the synergistic activation of PHOX2B with CBP [16]. Notably, Another family similar to our study demonstrated the impact of a PHOX2B gene mutation, resulting in symptomatic neuroblastoma in two out of three children. Strikingly, in this particular case, the mutation was inherited from the father, who subsequently developed a well-differentiated ganglioneuroma at age 40 [9]. However, In our presented family, the mother carrying the genetic variant does not show any symptoms, possibly due to tumor regression during childhood or the potential for tumor progression in adulthood.

In conclusion, this report identifies a novel mutation site in the PHOX2B gene and expands the spectrum of PHOX2B gene mutations. Families with a history of neuroblastoma should undergo genetic testing to determine the underlying cause and enable prenatal diagnosis, aiming to minimize the occurrence of congenital disabilities.

## Ethics approval and consent to participate

The procedures involving human patients were conducted under the approval of the Ethics Committee of the Affiliated Hospital of Qingdao University.

## Consent for publication

In this study, written informed consent has been obtained from the parents of the study participant. They have consented to the publication of identifying images or other personal or clinical details, and proof of consent can be provided upon request at any time.

## Funding

This research received no specific grant from any funding agency in the public, commercial, or not-for-profit sectors. The authors declare that they have no financial or non-financial competing interests.

## Data availability statement

Data will be made available on reasonable request.

## CRediT authorship contribution statement

**Xiongwei Wu:** Writing – original draft, Investigation, Conceptualization. **Wenli Xiu:** Supervision, Formal analysis, Conceptualization. **Na Zhou:** Writing – original draft, Resources, Formal analysis. **Jingli Zhang:** Writing – original draft, Supervision. **Xiwei Hao:** Supervision, Conceptualization. **Qian Dong:** Writing – review & editing, Conceptualization.

## Declaration of competing interest

The authors declare that they have no known competing financial interests or personal relationships that could have appeared to influence the work reported in this paper.

## References

[1] Zafar A., Wang W., Liu G. (2021). Molecular targeting therapies for neuroblastoma: progress and challenges. Med. Res. Rev..

[2] Tolbert V.P., Coggins G.E., Maris J.M. (2017). Genetic susceptibility to neuroblastoma. Curr. Opin. Genet. Dev..

[3] Mosse Y.P., Laudenslager M., Longo L. (2008). Identification of ALK as a major familial neuroblastoma predisposition gene. Nature.

[4] Zhao J., Zhu Y., Xie X. (2019). Pleiotropic effect of common PHOX2B variants in Hirschsprung disease and neuroblastoma. Aging (Albany NY).

[5] Maris J.M., Mosse Y.P., Bradfield J.P. (2008). Chromosome 6p22 locus associated with clinically aggressive neuroblastoma. N. Engl. J. Med..

[6] Capasso M., Devoto M., Hou C. (2009). Common variations in BARD1 influence susceptibility to high-risk neuroblastoma. Nat. Genet..

[7] Diskin S.J., Capasso M., Diamond M. (2014). Rare variants in TP53 and susceptibility to neuroblastoma. J. Natl. Cancer Inst..

[8] Stovroff M., Dykes F., Teague W.G. (1995). The complete spectrum of neurocristopathy in an infant with congenital hypoventilation, Hirschsprung's disease, and neuroblastoma. J. Pediatr. Surg..

[9] Bourdeaut F., Trochet D., Janoueix-Lerosey I. (2005). Germline mutations of the paired-like homeobox 2B (PHOX2B) gene in neuroblastoma. Cancer Lett..

[10] Amiel J., Laudier B., Attie-Bitach T. (2003). Polyalanine expansion and frameshift mutations of the paired-like homeobox gene PHOX2B in congenital central hypoventilation syndrome. Nat. Genet..

[11] Pattyn A., Hirsch M., Goridis C., Brunet J.F. (2000). Control of hindbrain motor neuron differentiation by the homeobox gene Phox2b. Development.

[12] Bachetti T., Di Paolo D., Di Lascio S. (2010). PHOX2B-mediated regulation of ALK expression: in vitro identification of a functional relationship between two genes involved in neuroblastoma. PLoS One.

[13] Kamihara J., Bourdeaut F., Foulkes W.D. (2017). Retinoblastoma and neuroblastoma predisposition and surveillance. Clin. Cancer Res..

[14] Trochet D., Bourdeaut F., Janoueix-Lerosey I. (2004). Germline mutations of the paired-like homeobox 2B (PHOX2B) gene in neuroblastoma. Am. J. Hum. Genet..

[15] van Limpt V., Schramm A., van Lakeman A. (2004). The Phox2B homeobox gene is mutated in sporadic neuroblastomas. Oncogene.

[16] Wu H.T., Su Y.N., Hung C.C. (2009). Interaction between PHOX2B and CREBBP mediates synergistic activation: mechanistic implications of PHOX2B mutants. Hum. Mutat..

